# A NKp80-Based Identification Strategy Reveals that CD56^neg^ NK Cells Are Not Completely Dysfunctional in Health and Disease

**DOI:** 10.1016/j.isci.2020.101298

**Published:** 2020-06-20

**Authors:** Ane Orrantia, Iñigo Terrén, Alicia Izquierdo-Lafuente, Juncal A. Alonso-Cabrera, Victor Sandá, Joana Vitallé, Santiago Moreno, María Tasias, Alasne Uranga, Carmen González, Juan J. Mateos, Juan C. García-Ruiz, Olatz Zenarruzabeitia, Francisco Borrego

**Affiliations:** 1 Biocruces Bizkaia Health Research Institute, Immunopathology Group, Barakaldo 48903, Spain; 2 Ramón y Cajal Health Research Institute (IRYCIS), Ramón y Cajal University Hospital, Madrid 28034, Spain; 3 Hospital Universitari i Politecnic La Fe, Valencia 46026, Spain; 4 Biodonostia Health Research Institute, Donostia University Hospital, Donostia-San Sebastián 20014, Spain; 5 Biocruces Bizkaia Health Research Institute, Hematological Cancer Group, Cruces University Hospital, Barakaldo 48903, Spain; 6 Ikerbasque, Basque Foundation for Science, Bilbao 48013, Spain

**Keywords:** Biological Sciences, Immunology

## Abstract

Natural killer (NK) cells are usually identified by the absence of other lineage markers, due to the lack of cell-surface-specific receptors. CD56^neg^ NK cells, classically identified as CD56^neg^CD16+, are very scarce in the peripheral blood of healthy people but they expand in some pathological conditions. However, studies on CD56^neg^ NK cells had revealed different results regarding the phenotype and functionality. This could be due to, among others, the unstable expression of CD16, which hinders CD56^neg^ NK cells’ proper identification. Hence, we aim to determine an alternative surface marker to CD16 to better identify CD56^neg^ NK cells. We have found that NKp80 is superior to CD16. Furthermore, we found differences between the functionality of CD56^neg^NKp80+ and CD56^neg^CD16+, suggesting that the effector functions of CD56^neg^ NK cells are not as diminished as previously thought. We proposed NKp80 as a noteworthy marker to identify and accurately re-characterize human CD56^neg^ NK cells.

## Highlights

• NKp80 is a more precise marker than CD16 in order to identify CD56^neg^ NK cells
• CD16, but no NKp80, is downmodulated after cryopreservation and cell activation
• CD56^neg^ NK cells effector functions are not as diminished as previously described

## Graphical Abstract

**Figure:**
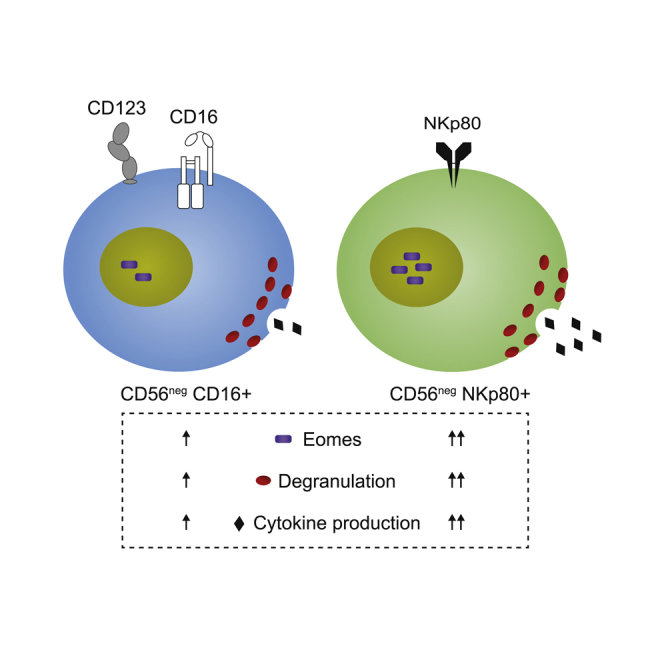

## Introduction

Natural killer (NK) cells are large granular lymphocytes that have the ability to recognize and kill transformed and virus-infected cells without prior sensitization (Caligiuri, 2008; Freud et al., 2017). In addition, they also produce and secrete a variety of cytokines and chemokines that modulate the immune response (Bancroft, 1993; Biron et al., 1999; Cooper et al., 2001; Robertson and Ritz, 1990). In human healthy adults, they comprise 5%–15% of circulating lymphocytes, being together with T cells and B cells, one of the three major lymphoid linages. However, in contrast to T and B cells, NK cells are a member of the innate lymphoid cells (ILCs) family, of which the main characteristic is the absence of rearranged antigen receptors encoded by the recombination activating genes (RAG) (Artis and Spits, 2015). Currently, ILCs are classified into five different subsets, depending on their effector functions, the cytokine pattern they secrete, and the transcription factors they need to develop and differentiate. The five subsets are NK cells, Group 1 ILC (ILC1), ILC2, ILC3, and lymphoid tissue-inducer (LTi) cells (Colonna, 2018; Vivier et al., 2018). NK cells and ILC1 are cells that produce interferon-γ (IFN-γ) as their signature cytokine and need the T-bet transcription factor to develop. On the other hand, ILC1 exhibit very little or no cytotoxic activity due to the low or zero levels of perforin and granzymes they express (Fuchs, 2016; Spits et al., 2016). Yet, the distinction between ILC1 and NK cells could be problematic because they express similar cell surface markers (Mjösberg and Spits, 2016; Spits et al., 2013; Trabanelli et al., 2018; Vivier et al., 2018). Nevertheless, in humans the NKp80 cell surface receptor is expressed on NK cells and seems to be an NK-cell-specific marker among human ILCs (Freud et al., 2016, 2017). Furthermore, NK cells express both T-bet and Eomesodermin (Eomes) transcription factors, whereas ILC1 only express T-bet (Artis and Spits, 2015; Bal et al., 2020; Colonna, 2018; Mjösberg and Spits, 2016; Spits et al., 2013; Vivier et al., 2018).

Commonly, as there is no known specific surface receptor that leads to NK cell identification within peripheral blood mononuclear cells (PBMCs), they are phenotypically defined as CD56+ cells that do not express other lineage markers, such as those that are specific for T cells (CD3), B cells (CD19), and myeloid cells (CD14). Furthermore, CD56 in combination with CD16, the low-affinity Fc gamma receptor IIIa, is generally used to distinguish different NK cell subsets that are present in healthy human peripheral blood (Freud et al., 2017; Montaldo et al., 2013). In this way, and based on the expression of these markers, two major subsets are identified: CD56^bright^CD16+/− and CD56^dim^CD16+. In addition, two more subsets have been described: CD56^neg^ (CD56^neg^CD16+) and unconventional CD56^dim^ (CD56+CD16−) NK cells (Hu et al., 1995; Lugli et al., 2014; Roberto et al., 2018; Di Vito et al., 2019). However, none of these markers are specific to NK cells. For instance, although all ILCs are also negative for the abovementioned linage markers (Spits et al., 2013; Trabanelli et al., 2018; Vivier et al., 2018), CD56 is expressed by a subset of ILC3, known as NCR+ ILC3 and which, as NK cells, expresses natural cytotoxicity receptors (NCR) NKp30, NKp44, and NKp46 (Spits et al., 2013; Trabanelli et al., 2018; Vallentin et al., 2015; Vivier et al., 2018). On the other hand, although CD16 is widely accepted to be an NK-cell-specific marker among ILCs (Spits et al., 2016; Trabanelli et al., 2018; Vivier et al., 2018), it is known that CD16 could be downregulated following target cell activation (Borrego et al., 1994; Grzywacz et al., 2007; Peruzzi et al., 2013; Romee et al., 2013; Zhou et al., 2013) and cryopreservation (Lugthart et al., 2015). Therefore, the lack of marker specificity makes the process of identifying NK cells quite challenging and highlights the need to start using other set of cell surface markers.

The CD56^neg^ subset expresses NK-cell-associated surface markers, such as CD16, CD94, and NKp46, in addition to the transcription factors Eomes and T-bet (Tarazona et al., 2002; Voigt et al., 2018). In healthy individuals, the presence of CD56^neg^ NK cells in blood is very rare (Björkström et al., 2010; Campos et al., 2014; Müller-Durovic et al., 2019). However, years ago, in patients with chronic human immunodeficiency virus (HIV)-1 infection, a significant expansion of CD56^neg^ NK cells was reported (Hu et al., 1995; Lugli et al., 2014), which was associated with high HIV-1 viral load (Alter et al., 2005; Barker et al., 2007; Mavilio et al., 2005). Indeed, long-term non-progressors and patients who successfully suppress viral load after highly active antiretroviral therapy have CD56^neg^ NK cells levels comparable to the ones found in non-infected subjects (Alter et al., 2005; Brunetta et al., 2009). However, patients who fail to suppress viral load upon treatment have similar CD56^neg^ numbers to the ones with persistent viremia (Alter et al., 2005). In addition, in chronically HIV-1-infected individuals who developed broadly neutralizing antibodies (bnAbs), a high proportion of NK cells have a CD56^neg^ phenotype, whereas in patients who do not have bnAbs the proportion of CD56^neg^ NK cells was lower, although still high when compared with HIV-1 seronegative subjects (Bradley et al., 2018). Elevated frequencies of the CD56^neg^ subset has also been described in hepatitis C virus (HCV)-monoinfected and HCV/HIV-1-coinfected people (Gonzalez et al., 2008, 2009). Furthermore, the abnormal expansion of these cells correlated with monoinfected patient's ability to respond to pegylated-IFNα and ribavirin treatment (Gonzalez et al., 2009). Moreover, treatments that suppress HCV replication decreases the number of CD56^neg^ NK cells in HCV-/HIV-1-coinfected patients (Gonzalez et al., 2008). On the other hand, it has been described that aging and human cytomegalovirus status has an effect on the frequency and distribution of NK cell subsets, increasing the percentage of CD56^neg^ NK cells (Campos et al., 2014; Müller-Durovic et al., 2019).

Studies with similar cohorts of patients differ in the frequency, functionality, and phenotype of the CD56^neg^ NK cell subset, which could be due to different gating strategies used for the identification of CD56^neg^ NK cells (Björkström et al., 2010; Eller et al., 2009; Mavilio et al., 2005; Milush et al., 2013). It is very important to note that the abovementioned studies, and many others, have identified this NK cell subset as CD56^neg^CD16+, and depending on the studies, they have or have not included in the gating strategy an exclusion channel for the exclusion of T cells, B cells, and/or monocytes within the cells of interest, i.e. CD56^neg^ NK cells. In addition, it is very well known that CD16 is downregulated by cryopreservation (Lugthart et al., 2015), after cytokine activation and target cell stimulation (Borrego et al., 1994; Grzywacz et al., 2007; Peruzzi et al., 2013; Romee et al., 2013; Zhou et al., 2013), and therefore the usage of this marker could lead to an inaccurate identification of the CD56^neg^ NK cells and inconsistent results. In this work, we have explored the possibility of using NKp80 as a marker to better identify CD56^neg^ NK cells. NKp80, the product of the *KLRF1* gene, is an activating receptor expressed by virtually all mature human NK cells (Vitale et al., 2001). NKp80 marks a critical step in NK cell development, as it defines functionally mature NK cells (Freud et al., 2016), and is an NK-cell-specific marker among human innate lymphoid cells (ILCs) (Vivier et al., 2018). We show that NKp80 is a more precise marker than CD16 in order to identify CD56^neg^ NK cells and that it is not downregulated after sample cryopreservation or cell activation. Importantly, using the NKp80 marker for the identification, we have demonstrated that the effector functions of CD56^neg^ NK cells are not as diminished as previously thought, both in health and in disease.

## Results

### The NKp80 Receptor Is Superior to CD16 for the Identification of Circulating CD56^neg^ NK Cell Subset in Healthy People

**Figure 1:**
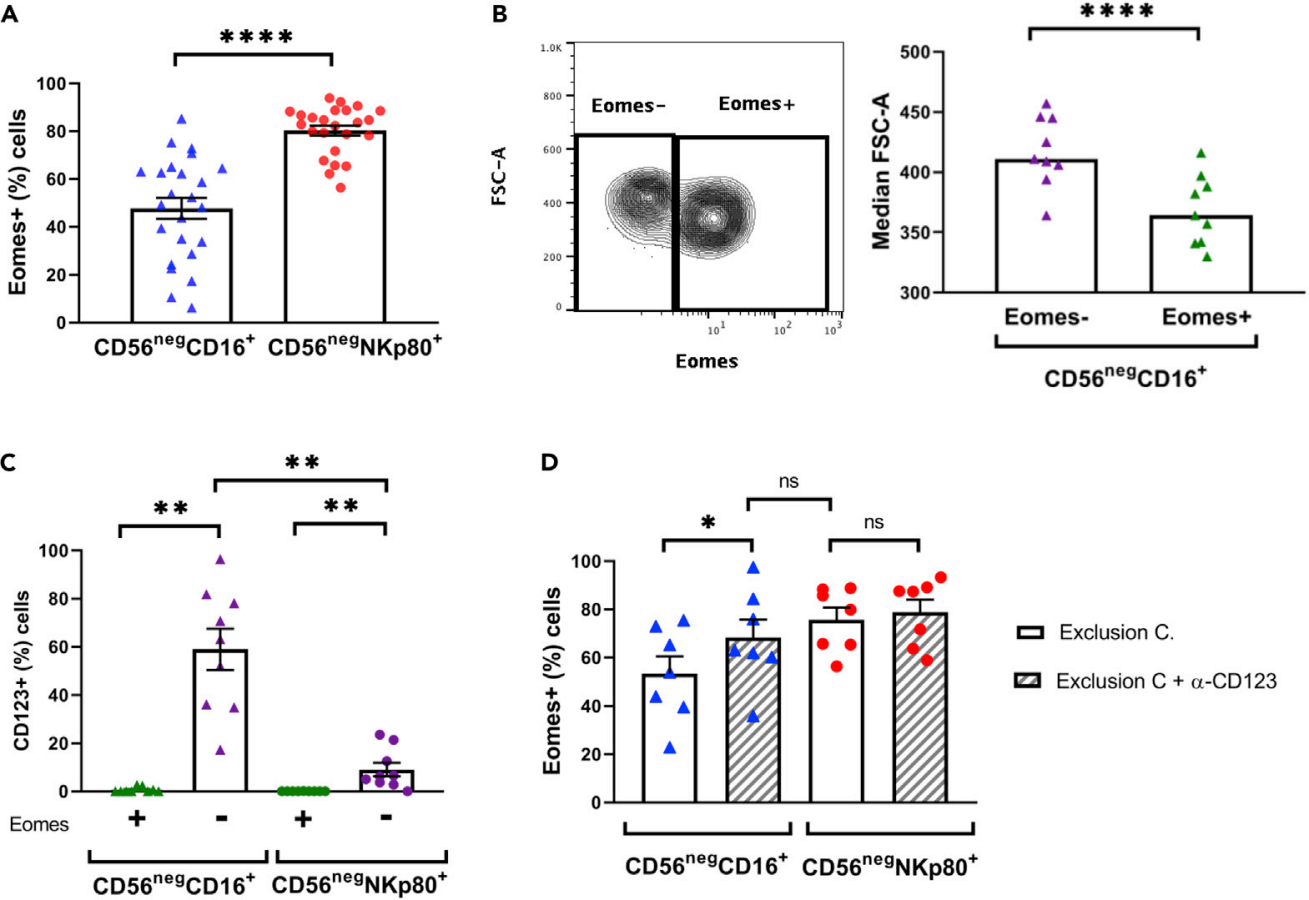
NKp80 Better Identifies CD56^neg^ NK Cells than CD16 in Healthy Individuals (A) Bar graph showing the percentage of Eomes+ cells within CD56^neg^CD16+ and CD56^neg^NKp80+ populations. (B) Left part, representative contour plot showing the Eomes expression versus the size (FSC-A) of CD56^neg^CD16+ cells. Data from a representative healthy donor is shown. Right part, bar graph showing the median of FSC-A parameter within CD56^neg^CD16+Eomes− and CD56^neg^CD16+Eomes+ populations. (C) Bar graph showing the percentage of CD123^+^ cells within CD56^neg^CD16+Eomes+, CD56^neg^CD16+Eomes−, CD56^neg^NKp80+Eomes+, and CD56^neg^NKp80+Eomes− populations. (D) Bar graph showing the percentage of Eomes+ cells within CD56^neg^CD16+ and CD56^neg^NKp80+ populations with or without the addition of anti-CD123 mAb to the exclusion channel (Exclusion C.). The mean with the standard error of the mean (SEM) is represented, except for (B) in which the median is represented. Each dot represents a donor. ∗p < 0.05, ∗∗p < 0.01, ∗∗∗∗p < 0.0001, ns: not significant.

The CD16 receptor has traditionally been used, in combination with CD56, to identify the circulating NK cell subsets, with CD56^neg^ NK cells defined as CD56^neg^CD16+ (Björkström et al., 2010). However, CD16 is well known to be downregulated in some situations, such as, cryopreservation, after target cell stimulation and compounds, cell surface receptor, and cytokine activation (Borrego et al., 1994; Grzywacz et al., 2007; Lugthart et al., 2015; Peruzzi et al., 2013; Romee et al., 2013; Zhou et al., 2013). CD16 is shed from the cell surface as a consequence of matrix metalloproteinases activation, such as MT6 (also known as MMP25) and ADAM17 (Grzywacz et al., 2007; Peruzzi et al., 2013; Romee et al., 2013). With the aim to identify a more accurate marker with a more stable expression, we first compared CD16 with NKp80 receptor to identify CD56^neg^ NK cells in healthy donors. Our gating strategy included an exclusion channel (viability, CD3, CD14, and CD19) that allowed us to specifically study non-T, non-B, non-monocytes viable cells (Figure S1A). As previously described (Lugthart et al., 2015), CD16 expression was downregulated in cryopreserved samples; however, the expression of NKp80 was not significantly altered after cell freezing (Figure S2), suggesting that this receptor is more suitable for the detection of CD56^neg^ NK cells when it concerns to frozen cells. Very importantly, although no differences were seen regarding the percentage of CD56^neg^ NK cells selected using both markers (Figure S1B), there was a significantly higher frequency of Eomes+ cells in the CD56^neg^NKp80+ subpopulation than in CD56^neg^CD16+ cells (Figure 1A). Eomes is a specific intracellular marker for the detection of NK cells within the ILCs, given that it is a T-box transcription factor needed for the development and function of NK cells, whereas for example, ILC1 do not express Eomes (Artis and Spits, 2015; Bal et al., 2020; Colonna, 2018; Mjösberg and Spits, 2016; Spits et al., 2013; Vivier et al., 2018). As the percentage of Eomes+ cells within the CD56^neg^CD16+ subset was low, we considered the possibility that other CD16+ non-NK cells could have been selected using this gating strategy. This hypothesis was strengthened by the fact that within the CD56^neg^CD16+ population, the Eomes^−^ cells had larger size than Eomes+ cells (Figure 1B). Thus, we studied the expression of CD123 receptor (α-chain of the interleukin 3 receptor) expressed, among others, in plasmacytoid dendritic cells (pDCs) and basophils, which are characterized by a larger size and granularity (Collin et al., 2013; Han et al., 2008; McKenna et al., 2005; Vitallé et al., 2019a; Zenarruzabeitia et al., 2019). Results showed that CD56^neg^CD16+Eomes− cells expressed CD123, in contrast to CD56^neg^NKp80+Eomes− cells that barely did (Figure 1C). Furthermore, the addition of an anti-CD123 mAb to the exclusion channel revealed that the frequency of CD56^neg^CD16+Eomes+ cells significantly increased but still tended to be lower compared with CD56^neg^NKp80+Eomes+ cells (Figure 1D). These results suggested that the inaccuracy in the identification of the CD56^neg^ NK cell subset using the CD16 marker in the gating strategy is due to the selection of Eomes− cells that, at least partially, could be pDCs and/or basophils, which are characterized by the expression of CD123.

**Figure 2:**
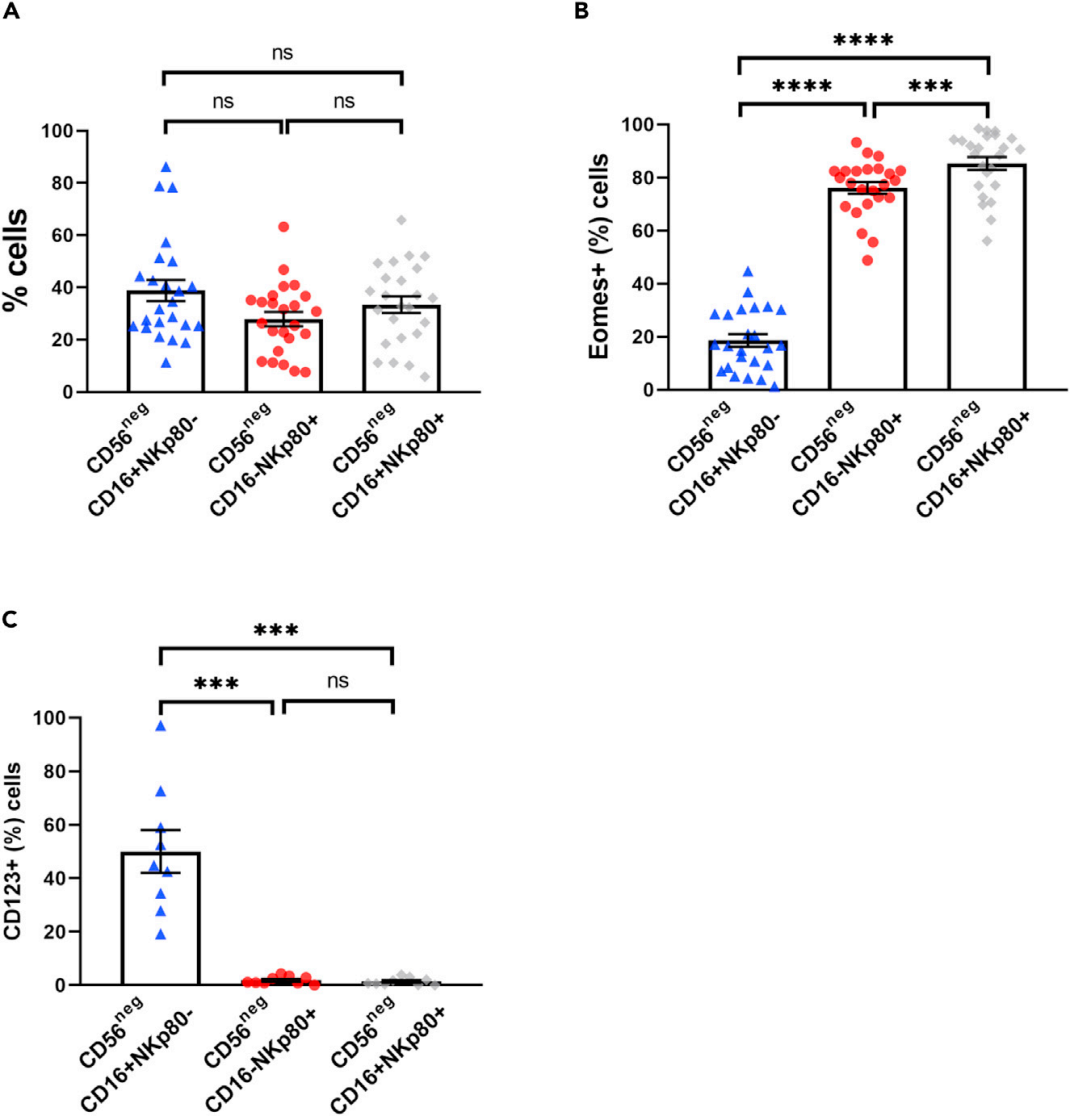
CD56^neg^CD16+NKp80− Subset Mostly Include Non-NK Cells (A) Bar graph showing the percentage of CD56^neg^CD16+NKp80−, CD56^neg^CD16−NKp80+ and CD56^neg^CD16+NKp80+ subsets within the CD56^neg^ cells. (B) Bar graph showing the percentage of Eomes+ cells within the CD56^neg^CD16+NKp80−, CD56^neg^CD16−NKp80+, and CD56^neg^CD16+NKp80+ populations. (C) Bar graphs showing the percentage of CD123+ cells within the CD56^neg^CD16+NKp80−, CD56^neg^CD16−NKp80+, and CD56^neg^CD16+NKp80+ populations. The mean with the standard error of the mean (SEM) is represented. Each dot represents a donor. ∗∗∗p < 0.001, ∗∗∗∗p < 0.0001, ns: not significant.

Given that there are no significant differences in the frequency of CD56^neg^CD16+ and CD56^neg^NKp80+ cells (Figure S1B), but the latter expressed significantly higher levels of Eomes (Figure 1A), we analyzed if NKp80 is inclusive of the CD56^neg^CD16+ subset. Results showed no significant differences in the frequency of CD16+NKp80−, CD16−NKp80+, and CD16+NKp80+ subsets within the CD56^neg^ cells and that half of the CD56^neg^NKp80+ NK cells also co-express CD16 (Figure 2A). Importantly, when the expression of Eomes was analyzed within these three subsets, we found that the frequency of Eomes+ cells was very low in CD16+NKp80− cells and significantly higher in both CD16−NKp80+ and CD16+NKp80+ cells (Figure 2B). Moreover, although CD16+NKp80− cells included a significant frequency of CD123+ cells (50%), both CD16−NKp80+ and CD16+NKp80+ subsets comprised negligible levels of CD123+ cells (Figure 2C). Altogether, these results suggest that although CD16 and NKp80 do not completely identify the same CD56^neg^ cells, NKp80 is more precise for the identification of CD56^neg^ NK cells.

**Figure 3:**
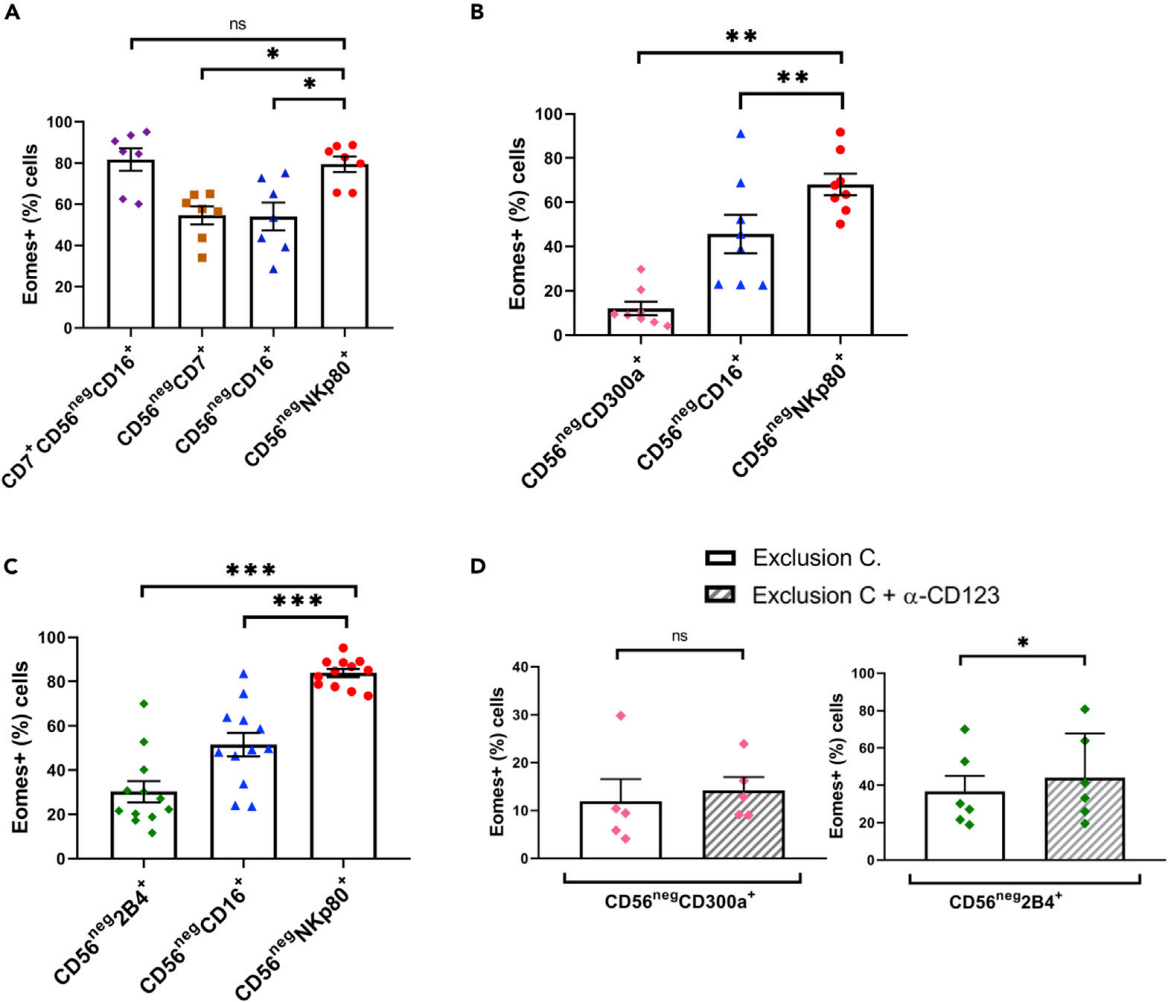
NKp80 Better Identifies CD56^neg^ NK Cells than CD7, CD300a, and 2B4 (CD244) in Healthy Individuals (A) Bar graph showing the percentage of Eomes+ cells within CD7+CD56^neg^CD16+, CD56^neg^CD7+, CD56^neg^CD16+, and CD56^neg^NKp80+ populations. (B) Bar graph showing the percentage of Eomes+ cells within CD56^neg^CD300a+, CD56^neg^CD16+, and CD56^neg^NKp80+ populations. (C) Bar graph showing the percentage of Eomes+ cells within CD56^neg^2B4+, CD56^neg^CD16+, and CD56^neg^NKp80+ populations. (D) Bar graphs showing the percentage of Eomes+ cells within CD56^neg^CD300a+ and CD56^neg^2B4+ populations with or without the addition of anti-CD123 mAb to the exclusion channel (exclusion C.). The mean with the standard error of the mean (SEM) is represented. Each dot represents a donor. ∗p < 0.05, ∗∗p < 0.01, ∗∗∗p < 0.001, ns: not significant.

More recently, it was shown that including CD7 as an additional marker to the CD56^neg^CD16+ cell subset was an effective method to accurately identify CD56^neg^ NK cells (Milush et al., 2009, 2013). Therefore, we compared the frequency of Eomes+ cells using CD7 or NKp80 markers to identify the CD56^neg^ NK cell subpopulation. There were no significant differences between CD7+CD56^neg^CD16+ and CD56^neg^NKp80+ cells in terms of Eomes expression, indicating that both strategies were equally effective for the identification of CD56^neg^ cells in these specific experimental settings. However, when we only used the CD7 marker instead of NKp80 the frequency of Eomes+ cells in the CD7+CD56^neg^ population was much lower than in both CD7+CD56^neg^CD16+ and CD56^neg^NKp80+ cells (Figure 3A). On the other hand, combining the CD7 and CD16 markers to identify the CD56^neg^ NK cell subset could be an obstacle in certain situations, especially due to the unstable expression of the CD16 receptor after cryopreservation and cell stimulation, and also the need for an additional monoclonal antibody to the panel and an extra flow cytometer detector, which could be overcome by only using NKp80 as a marker for the identification of CD56^neg^ NK cells.

Next, we studied the CD300a (Dimitrova et al., 2016; Vitallé et al., 2019b; Zenarruzabeitia et al., 2016) and 2B4 (CD244) (Endt et al., 2007) receptors that are also expressed in NK cells, although not exclusively, as markers for the identification of the CD56^neg^ NK cells. Results showed a lower frequency of Eomes+ cells both in CD56^neg^CD300a+ and in CD56^neg^2B4+ cells compared with CD56^neg^NKp80+ cells (Figures 3B and 3C). Moreover, the addition of an anti-CD123 mAb to the exclusion channel minimally increased the frequency of Eomes+ cells (Figure 3D). Altogether, our results demonstrate that NKp80 is the best cell surface marker to identify the CD56^neg^ NK cell subset with a very high certainty and accuracy.

### CD56^neg^NKp80+ Cells Are Expanded in HIV-Infected People and Patients with Multiple Myeloma

**Figure 4:**
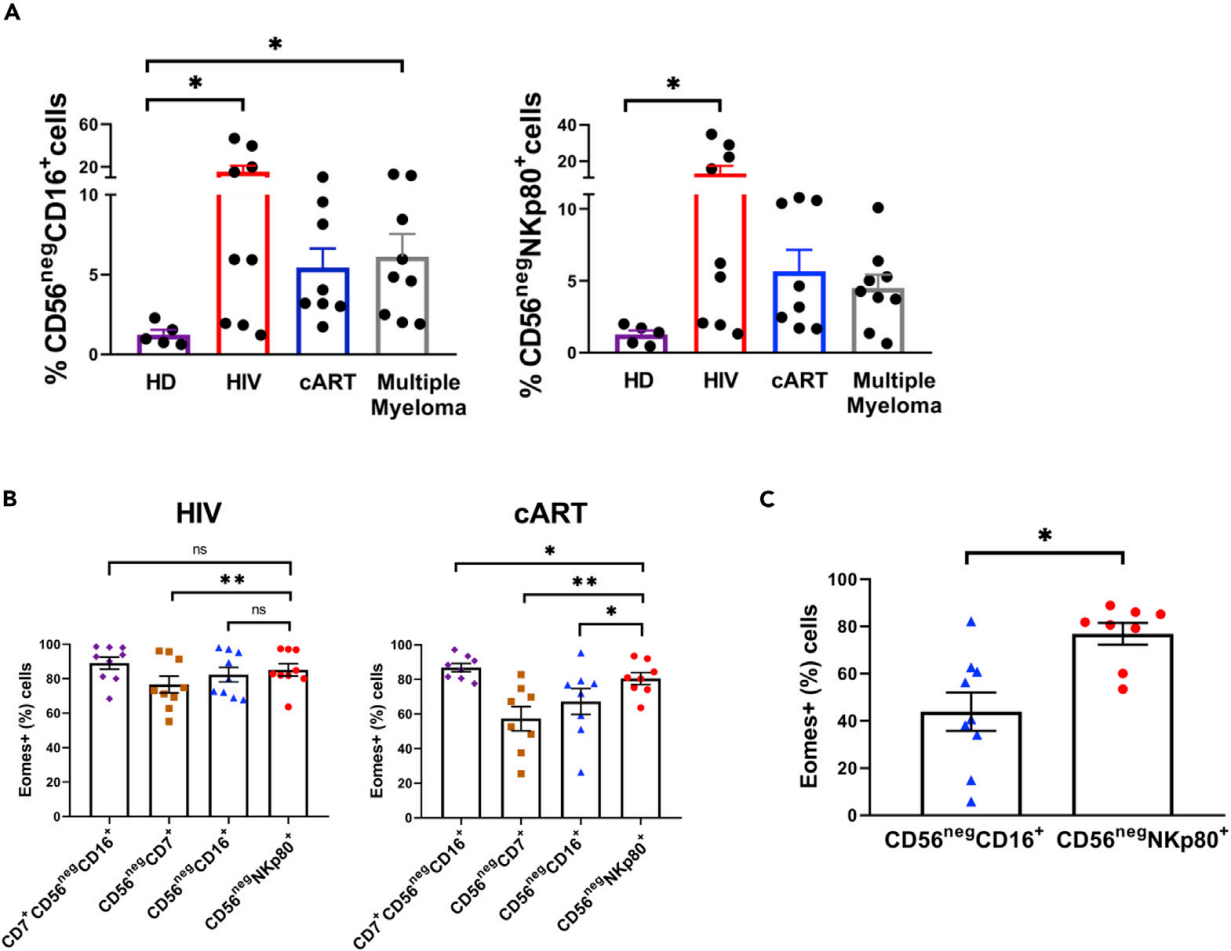
NKp80 Better Identifies CD56^neg^ NK Cells than CD16 in Pathological Conditions (A) Bar graphs showing the percentage of CD56^neg^CD16+ (left part) and CD56^neg^NKp80+ (right part) subsets within total NK cells from healthy donors (HD), untreated HIV-1 infected people (HIV), HIV-1-infected patients under cART (cART) and multiple myeloma patients. (B) Bar graphs showing the percentage of Eomes+ cells within CD7+CD56^neg^CD16+, CD56^neg^CD7+, CD56^neg^CD16+, and CD56^neg^NKp80+ populations in untreated HIV-1-infected subjects (HIV) and HIV-1-infected patients under cART (cART). (C) Bar graph showing the percentage of Eomes+ cells within CD56^neg^CD16+ and CD56^neg^NKp80+ populations in multiple myeloma patients. The mean with the standard error of the mean (SEM) is represented. Each dot represents a donor. ∗p < 0.05, ∗∗p < 0.01, ns: not significant.

As CD56^neg^ NK cells are infrequent in the peripheral blood of healthy donors, we next evaluated the accuracy of the NKp80 receptor to identify the expanded CD56^neg^ NK cells in pathological conditions, such as HIV infection and multiple myeloma (Figure 4A; Table S1). No significant differences were noticed in the frequency of Eomes+ cells between CD56^neg^CD16+ and CD56^neg^NKp80+ subpopulations in untreated HIV-1 infected subjects. However, the frequency of Eomes+ cells was significantly higher in CD56^neg^NKp80+ than in CD56^neg^CD16+ cells in HIV-1 infected subjects under combined antiretroviral therapy (cART) (Figure 4B). The differences between patient groups could be explained because the relative frequency of CD56^neg^CD16+ non-NK cells (Eomes−) is lower in untreated patients due to a higher expansion of the CD56^neg^CD16+ NK cells (Eomes+) (Alter et al., 2005; Barker et al., 2007; Mavilio et al., 2005). Thus, CD16 could only serve to identify CD56^neg^ NK cells in certain pathological conditions in which this subset is very highly expanded*.* In addition, a higher frequency of Eomes+ cells within CD56^neg^NKp80+ cells in comparison with CD56^neg^CD16+ cells was also noticeable in multiple myeloma patients (Figure 4C), in which CD56^neg^ NK cell expansion is more similar to the one of HIV-1 infected subjects under cART (Figure 4A). These findings suggest that NKp80, as demonstrated in healthy donors, is a noteworthy alternative to CD16 as a marker to identify CD56^neg^ NK cells also in disease.

### CD56^neg^NKp80+ Cells Are More Functional than CD56^neg^CD16+ Cells

Although surface receptor profiling and proteomic analyses indicate that CD56^neg^ have a phenotypic relationship to CD56^dim^ (Voigt et al., 2018), CD56^neg^ NK cells have been described as functionally impaired compared with CD56^dim^ NK cells (Alter et al., 2005; Björkström et al., 2010; Mavilio et al., 2005; Milush et al., 2013). However, others have proposed that these cells are skewed rather than dysfunctional (Eller et al., 2009). These differences may be due to inaccurate identification of CD56^neg^ NK cells using the CD16 marker. Therefore, we studied the effector functions of CD56^neg^CD16+ and CD56^neg^NKp80+ cells from healthy donors and HIV-1 infected subjects, by measuring degranulation (CD107a) (Alter et al., 2004) and production of TNF and IFNγ after cytokines and K562 target cell stimulations (Terrén et al., 2018).

**Figure 5:**
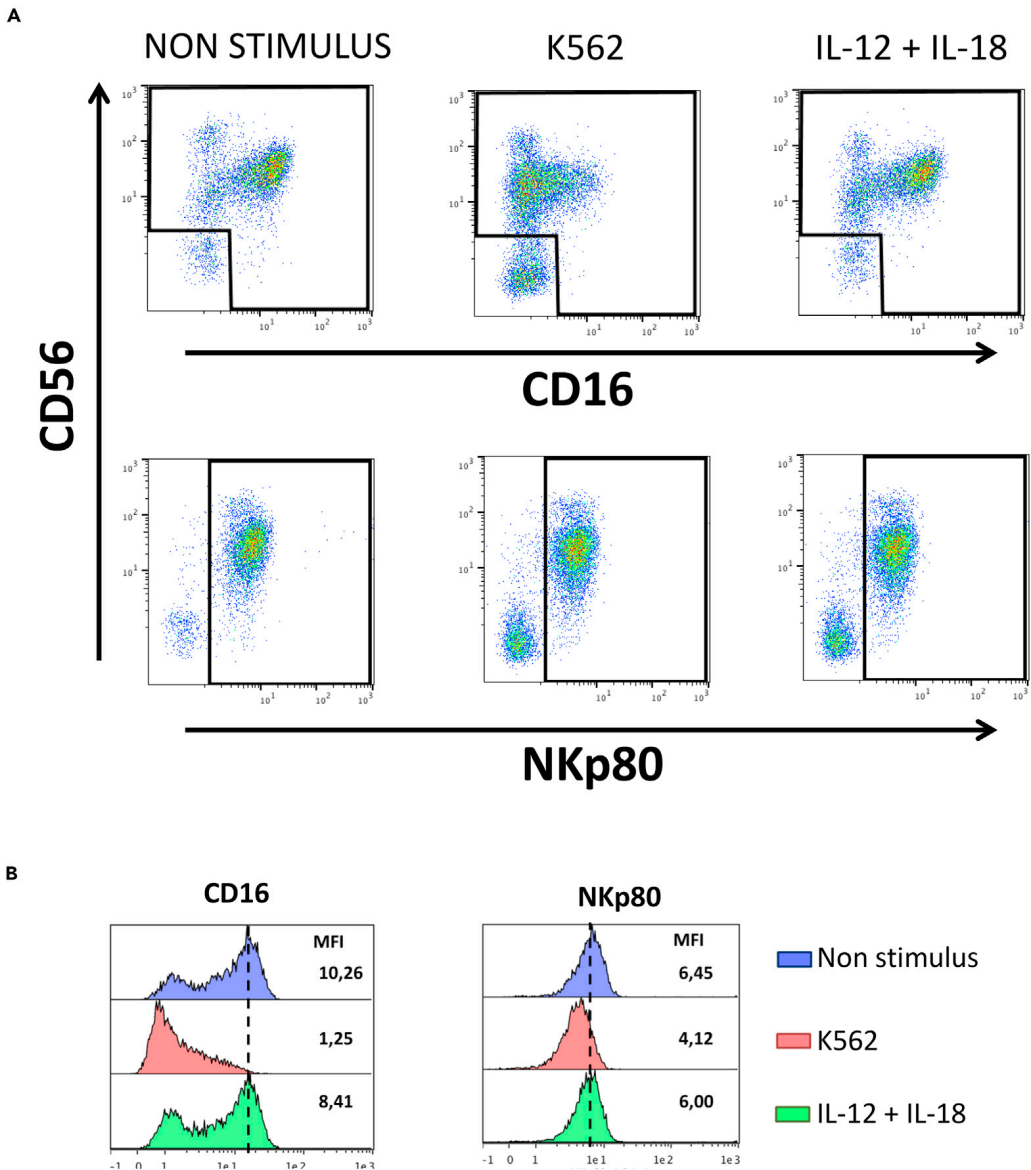
CD16, But no NKp80, Is Downregulated after K562 Cell Line and IL-12+IL-18 Cytokine Stimulation (A) Representative pseudocolor plot graphs comparing the expression of CD16 and NKp80 in non-stimulated condition, and after K562 cell line and IL-12+IL-18 cytokine stimulation. Data from a representative healthy donor is shown. (B) Histograms showing the median fluorescence intensity (MFI) of CD16 and NKp80 on NK cells in non-stimulus condition and after K562 cell line and IL-12+IL-18 cytokine stimulation. Data from a representative healthy donor is shown.

**Figure 6:**
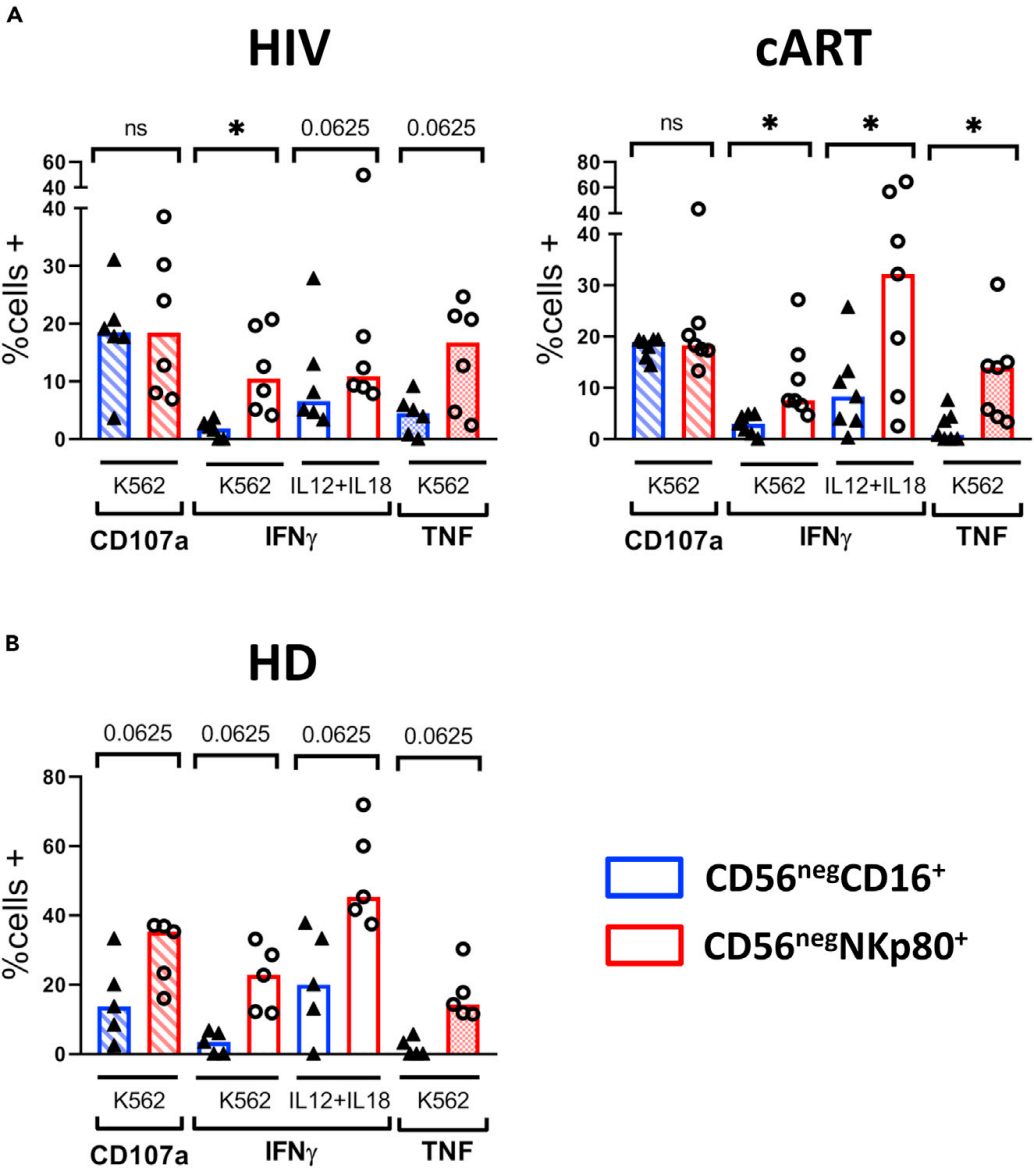
Degranulation (CD107a) and Cytokine Production (IFNγ and TNF) by CD56^neg^ NK Cells in Response to K562 Cell Line and IL-12+IL-18 Stimulation (A) Bar graphs showing the percentage of positive cells for CD107a, IFNγ, and TNF from HIV-1-infected subjects (HIV) and HIV-1-infected patients under cART (cART) after stimulation with the K562 cell line and IL-12+IL-18 cytokines within CD56^neg^CD16+ and CD56^neg^NKp80+ NK cells. (B) Bar graph showing the percentage of positive cells for CD107a, IFNγ, and TNF from healthy donors (HD) after stimulation with the K562 cell line and IL-12+IL-18 cells within CD56^neg^CD16+ and CD56^neg^NKp80+ NK cells. The median is represented. Each dot represents a donor. ∗p < 0.05, ns: not significant.

First, we wanted to compare CD16 and NKp80 expression downregulation after NK cell stimulation. Results showed that NKp80 was not significantly downregulated after K562 cell line and cytokine stimulation, whereas CD16 expression significantly decreased after stimulation with both stimuli (Figures 5A and 5B). In terms of functionality, we observed that CD56^neg^NKp80+ cells exhibited higher production of TNF and IFNγ than CD56^neg^CD16+ cells in treated HIV-1 infected subjects and that they showed a tendency to a higher production of both cytokines in untreated subjects (Figure 6A). Furthermore, in healthy donors, both cytokine production and the degranulation capability tended to be higher in the CD56^neg^NKp80+ cells than in CD56^neg^CD16+ cells (Figure 6B). Finally, we compared the functionality of the CD56^dim^ and CD56^neg^ NK cells. Very importantly, our results showed that, although CD56^neg^ NK cells have lower effector functions than CD56^dim^ NK cells (Figure S3) in healthy donors and HIV-infected people, their functionality is much lower when we used the CD16-based gating strategy to identify CD56^neg^ NK cells than when we used the NKp80-based gating strategy. Altogether, our results indicate that CD56^neg^ NK cells, defined as viable CD3-CD19-CD14-CD56^neg^NKp80+ cells, are significantly less dysfunctional than previously thought.

## Discussion

It is of utmost importance to correctly phenotype the different subpopulations of immune cells not only in healthy people but also in disease situations. In the latter, variations in the frequency of cells subsets and in their effector functions, in comparison to healthy state, are frequently observed. These variations can help to understand the pathogenesis of diseases, and moreover, these changes can serve as biomarkers for the diagnosis, prognosis, and/or to determine the efficacy of the treatment.

Some immune cell types are characterized by the expression of specific lineage cell surface markers. For example, T cells are CD3+, whereas other cell types do not express CD3. However, a specific NK cell surface marker has not been described yet. In general terms, the minimum requirement to define circulating human NK cells is based on the expression of CD56 and the absence of the CD3 marker, because an important subpopulation of T cells expresses CD56 (Ortaldo et al., 1991). However, there are other cells, such as ILC3, which can also express CD56 (Artis and Spits, 2015; Spits et al., 2013; Vallentin et al., 2015; Vivier et al., 2018). On the other hand, according to the expression of CD56 and CD16, NK cells have been classified into four subpopulations: CD56^bright^ (CD56^bright^CD16+/−), CD56^dim^ (CD56^dim^CD16+), unconventional CD56^dim^ (CD56^dim^CD16−), and CD56^neg^ (CD56^neg^CD16+) (Freud et al., 2017; Hu et al., 1995; Montaldo et al., 2013; Roberto et al., 2018; Di Vito et al., 2019). Finally, although the Eomes transcription factor is also expressed in CD4+ and CD8+ T cells (Knox et al., 2014; Narayanan et al., 2010), it is used as a specific intracellular marker of NK cells within the ILCs.

CD56^neg^ cells represent a very low percentage of NK cells in peripheral blood from healthy people (Björkström et al., 2010; Campos et al., 2014; Müller-Durovic et al., 2019). However, in certain diseases there is a very significant expansion of this cell subpopulation (Alter et al., 2005; Barker et al., 2007; Forconi et al., 2018; Hu et al., 1995; Lugli et al., 2014; Mavilio et al., 2005). To our knowledge, in previous publications, the expression of the CD16 marker have so far been used in all the gating strategies to identify the CD56^neg^ NK cell subset. Some authors have made use of a strategy based on only three markers (CD3, CD56, and CD16) (Alter et al., 2005, 2006; Barker et al., 2007; Campos et al., 2014; Frias et al., 2015; Jiang et al., 2011; Lu et al., 2008; Zarife et al., 2009). This strategy poses the risk of including other cell types that are not NK cells in the CD56^neg^ subpopulation, such as non-classical monocytes, which express high levels of CD16 (Ong et al., 2019; Ziegler-Heitbrock et al., 2010). Other authors have used an exclusion channel that includes other lineage markers in addition to CD3 (T cells), such as CD19 (B cells), CD14 (monocytes), and CD4 (T cells and monocytes), so these cell types will not be included within the CD56^neg^ cells (Bradley et al., 2018; Eller et al., 2009; Gonzalez et al., 2009; Jacobson et al., 2013; Jia et al., 2013; Nabatanzi et al., 2019; Rao et al., 2018; Zulu et al., 2017). However, even using these exclusion channels for the identification of the CD56^neg^ subpopulation, other cells that are not NK cells could be included, such as basophils and pDCs, that are characterized, among others, by the expression of CD123 (Collin et al., 2013; Han et al., 2008; McKenna et al., 2005; Vitallé et al., 2019a). In fact, our results demonstrate that adding an anti-CD123 mAb to the exclusion channel significantly increases the number of NK cells (Eomes+) within the CD56^neg^ subpopulation (Figure 1). A progress to better identify CD56^neg^ NK cells consists in a gating strategy that, in addition to the exclusion channel (CD3, CD14 and CD19), includes the lymphocyte specific marker CD7, which among other things, showed a high Eomes expression (Müller-Durovic et al., 2019; Voigt et al., 2018). However, it is important to note that CD7 is also expressed and used to identify ILC2 (Lim et al., 2016). Very significantly, our data demonstrate that the percentage of NK cells (Eomes+) is the same in CD7+CD56^neg^CD16+ cells and in CD56^neg^NKp80+ cells (Figure 3). However, our gating strategy needs to add one less antibody to the panel and the use of one less detector in the flow cytometer, which can be very relevant in many laboratories that do not have such advanced and sophisticated equipment.

The cryopreservation of biological samples is very advantageous in certain circumstances, such as when the samples are collected in locations far from the place where cytometry studies are performed. Cryopreservation also provides an additional benefit, since samples can be analyzed in large batches, minimizing overall analytical variability. This is especially important for studies in which the collection occurs over a long period of time or a large number of samples are obtained. However, cryopreservation also has drawbacks. It is well known that the expression of certain receptors, including CD16, is downregulated with freezing (Lugthart et al., 2015) (Figure S2). Therefore, when frozen samples are used, the gating strategy for the identification of CD56^neg^ NK cells based on CD16 expression is probably not the most appropriate, thus, the results obtained on studies that have been conducted with cryopreserved samples (Campos et al., 2014; Müller-Durovic et al., 2019; Nabatanzi et al., 2019; Pohlmeyer et al., 2019; Zulu et al., 2017) should be carefully considered. Following this, we have shown that the expression of NKp80 is not significantly modulated during cryopreservation (Figure S2). Therefore, NKp80 is a suitable marker for the study of CD56^neg^ NK cells in frozen samples. However, NKp80 is not a specific marker of NK cells. In fact, a minor subset of CD8+ T cells also express it (Kuttruff et al., 2009). Nevertheless, using an exclusion channel that includes CD3, such as the one we used in this study, these NKp80+CD8+ T cells will be not selected within the CD56^neg^ NK cell subset.

Finally, it has been documented for a long time that CD16 expression is downregulated after activation of NK cells with cytokines, target cells, antibody-dependent-cell-mediated cytotoxicity (ADCC) and after stimulation with compounds such as PMA (Borrego et al., 1994; Peruzzi et al., 2013; Romee et al., 2013; Zhou et al., 2013). Therefore, a gating strategy based on CD16 expression to analyze the effector functions of the CD56^neg^ NK cells is not very appropriate, especially in those situations when the stimuli, such as PMA and ADCC, induce a very deep downmodulation of CD16 (Borrego et al., 1994; Harrison et al., 1991; Romee et al., 2013; Zhou et al., 2013). Thus, the results of functional studies of CD56^neg^ NK identified with a CD16-based strategy should also be carefully considered (Alter et al., 2005; Eller et al., 2009; Milush et al., 2013; Nabatanzi et al., 2019). Related to NKp80, Klimosch et al. have described that NK cells stimulated with PMA or IL-2+IL-12+IL-18 induced the downregulation of this receptor after 24 h of stimulation (Klimosch et al., 2013). However, in our hands, and in response to stimulation with IL-15+K562 target cells and IL-15+IL-12+IL-18, the expression of NKp80 is not significantly altered (Figure 5). This difference could be due to, among others, the different cell stimulation protocols used in both studies. Thus, we propose NKp80 as a suitable marker for the study of the effector functions of CD56^neg^ NK cells. In fact, our results show that when we use NKp80 in the gating strategy, CD56^neg^ cells have greater effector functions compared with CD56^neg^ cells identified with CD16 (Figure 6).

In conclusion, in this study we have demonstrated that NKp80 is a more precise marker than CD16 in order to identify CD56^neg^ NK cells and that it is not downregulated after sample cryopreservation or cell activation. Importantly, using the NKp80 marker for the identification, we have demonstrated that the effector functions of CD56^neg^ NK cells are not as diminished as previously thought. Thus, an NKp80-based strategy for the better identification and re-characterization of this NK cell subset could help to clarify its function and relevance in health and disease.

### Limitation of the Study

In this study we proposed that an NKp80-based gating strategy will significantly improve the identification of circulating CD56^neg^ NK cells compared with a gating strategy based on CD16. This is even more relevant when cryopreserved samples are used and functional studies are performed. However, we have to consider that NKp80 is not a specific marker of NK cells because some CD8+ T cells also express it, although these cells are gated out by using an exclusion channel. Maybe more important is that a small percentage of CD56^neg^NKp80+ cells (around %20) does not express Eomes (Figure 1A). Thus, although NKp80 is not the perfect surface marker to identify the intriguing CD56^neg^ NK cell subset, it is the best currently available. Therefore, our study is a noteworthy step forward in the right direction in the field.

### Resource Availability

#### Lead Contact

Further information and request for resources and reagents should be directed to and will be fulfilled by the Lead Contact, Francisco Borrego (francisco.borregorabasco@osakidetza.eus).

#### Materials Availability

This study did not generate new unique reagents.

#### Data and Code Availability

This study did not generate datasets and codes.

## Methods

All methods can be found in the accompanying Transparent Methods supplemental file.

## Supplemental Information

Document S1. Transparent Methods, Figures S1–S3, and Table S1
