# Supplementary material for: A NKp80-Based Identification Strategy Reveals that CD56neg NK Cells Are Not Completely Dysfunctional in Health and Disease
Source: iScience. 2020 Jun 20;23(7):101298. doi: 10.1016/j.isci.2020.101298 (PMC7334412; doi:10.1016/j.isci.2020.101298)
Supplement: Document S1. Transparent Methods, Figures S1–S3, and Table S1 [file mmc1.pdf]

iScience, Volume *23*

Supplemental Information

A NKp80-Based Identification Strategy Reveals that CD56^{neg} NK Cells Are Not Completely Dysfunctional in Health and Disease

Ane Orrantia, Iñigo Terrén, Alicia Izquierdo-Lafuente, Juncal A. Alonso-Cabrera, Victor Sandá, Joana Vitallé, Santiago Moreno, María Tasias, Alasne Uranga, Carmen González, Juan J. Mateos, Juan C. García-Ruiz, Olatz Zenarruzabeitia, and Francisco Borrego

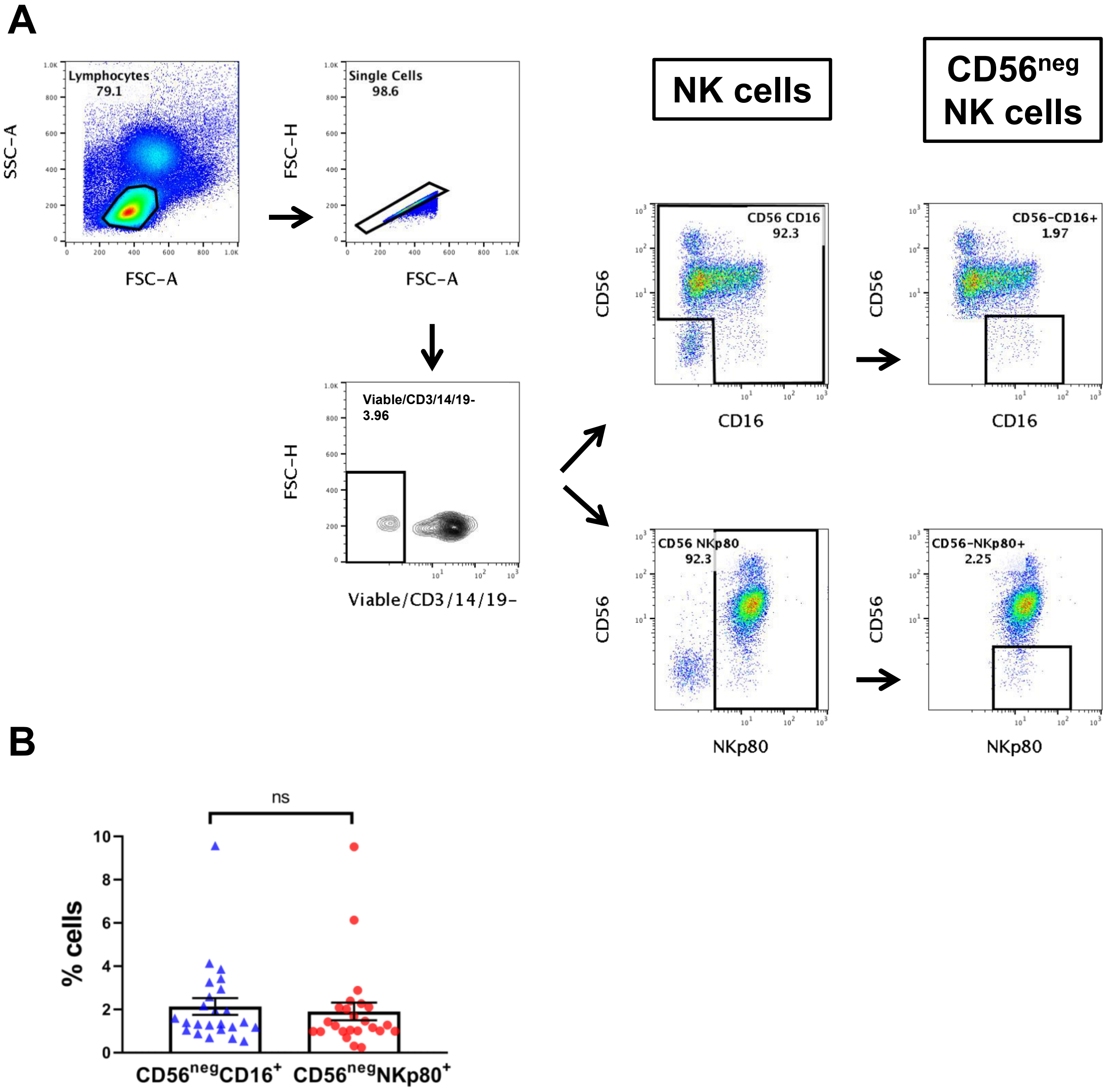

Figure S1. Identification of CD56neg NK cells. Related to Figure 1**. (A)** Pseudocolor and contour plot graphs representing the gating strategy utilized for the identification of CD56^{neg} NK cells. Data from a representative cryopreserved sample from a healthy donor is shown. Lymphocytes were electronically gated based on their forward and side scatter parameters and then single cells were selected. To identify NK cells, the population negative for the exclusion channel (viability, CD3, CD14 and CD19) was selected. Then CD56^{neg} NK cells were identified using different gating strategies. **(B)** Percentage of CD56^{neg}CD16+ and CD56^{neg}NKp80+ cells in healthy donors. Bar graph showing the percentage of CD56^{neg}CD16+ and CD56^{neg}NKp80+ cells in healthy donors. The mean with the standard error of the mean (SEM) is represented. Each dot represents a donor. ns: not significant.

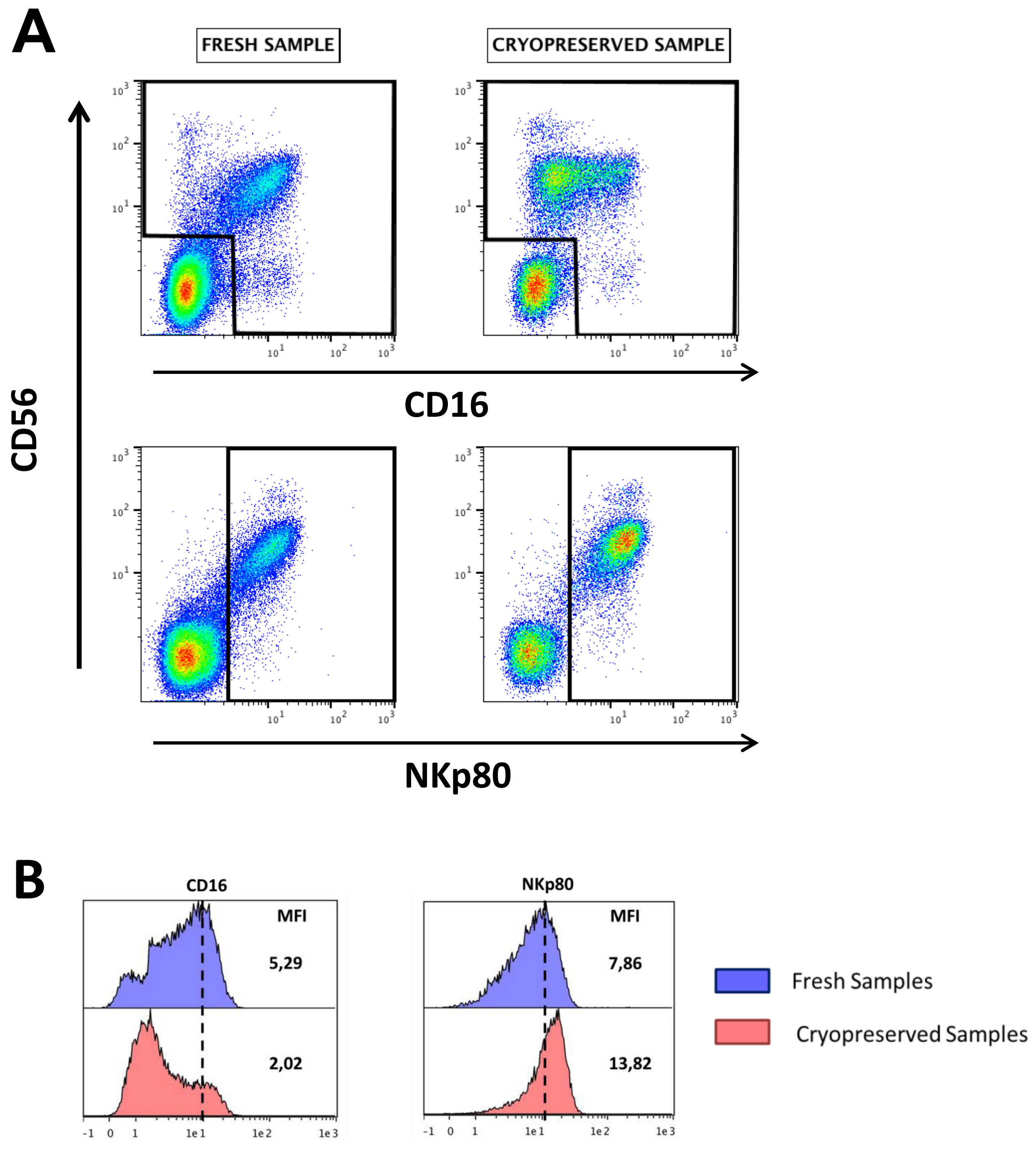

Fig. S2. CD16 but not NKp80 is downregulated after cell cryopreservation. Related to Figure 1. **(A)** Representative pseudocolor plot graphs comparing the expression of CD16 and NKp80 in fresh and cryopreserved samples. Data from a representative healthy donor is shown. **(B)** Histograms showing the median fluorescence intensity (MFI) of CD16 and NKp80 on NK cells in fresh and cryopreserved samples. Data from a representative healthy donor is shown.

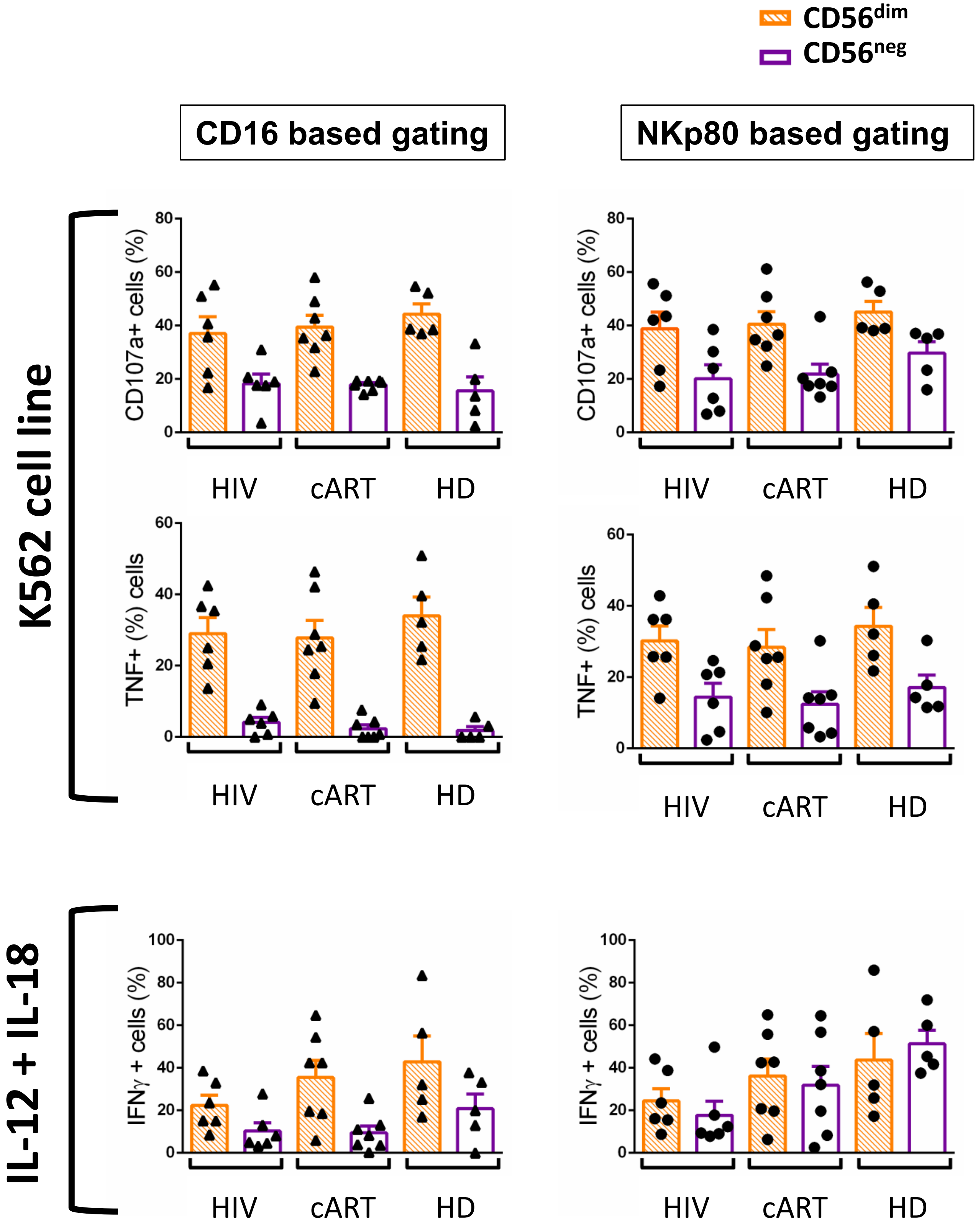

Fig. S3. Degranulation (CD107a) and cytokine production by NK cells in response to the K562 cell line and IL-12+IL-18 cytokine stimulation. Related to Figure 6**.** Bar graphs showing the percentage of CD56^{dim} and CD56^{neg} NK cells positive for CD107a and TNF after K562 cell line stimulation and IFNγ after IL-12+IL-18 cytokine stimulation from HIV-1 infected subjects (HIV), HIV-1 infected patients under cART (cART) and healthy donors (HD). The mean with the standard error of the mean (SEM) is represented. Each dot represents a donor.

Table S1. Clinical data of untreated HIV-1 infected subjects, under cART HIV-1 infected patients and multiple myeloma patients. Related to Figure 4 and Figure 6.

	**Untreated HIV-1 subjects**		**HIV-1 patients on cART**		**Multiple myeloma patients**	
	**Median**	**Range (min-max)**	**Median**	**Range (min-max)**	**Median**	**Range (min-max)**
**Sex**	Female: n=0<br>Male: n=9	-	Female: n=1<br>Male: n=7	-	Female: n=6<br>Male: n=3	-
**Age (years)**	39	(32-59)	49	(34-56)	63	(53-74)
**cART (months)**	-	-	13	(10-27)	-	-

TRANSPARENT METHODS

Contact for reagents and resource sharing

Further information and request for resources and reagents should be directed to and will be fulfilled by the Lead Contact, Francisco Borrego (francisco.borregorabasco@osakidetza.eus). This study did not generate new unique reagents.

Experimental Model and Subject Details

For this study, buffy coats from 24 healthy adult donors and cryopreserved peripheral blood mononuclear cells (PBMCs) from 9 multiple myeloma patients were collected through the Basque Biobank for Research (http://www.biobancovasco.org), which complies with the quality management, traceability and biosecurity, set out in the Spanish Law 14/2007 of Biomedical Research and in the Royal Decree 1716/2011. The study was approved by the Basque Ethics Committee for Clinical Research (PI2014017 and PI+CES+INC-BIOEF 2017-03). All subjects provided written and signed informed consent in accordance with the Declaration of Helsinki. In addition, cryopreserved PBMCs from healthy donors (n=5), untreated HIV-1 infected subjects (n=9) and patients under cART (n=8) were provided by the HIV BioBank integrated in the Spanish AIDS Research Network (RIS) (Supplementary Information, Appendix I). Samples were processed following current procedures and frozen immediately after their reception. All patients participating in the study gave their informed consent and protocols were approved by institutional ethical committees.

All HIV-1 infected patients were asymptomatic when the sample was collected, were not co-infected with hepatitis C virus (HCV), had more than 200 CD4+ T cells/mm^3 and they had never been diagnosed with AIDS. Untreated HIV-1 infected subjects had detectable viremia (>10,000 HIV-RNA copies/ml) and they had never been treated with cART, while patients under cART had undetectable viremia and had been treated with cART at least for 6 months. Clinical data of HIV-1 infected patients were obtained from the RIS database. Clinical data are shown in Table S1.

Antibodies and reagents

For flow cytometry-based procedures, the following fluorochrome-conjugated anti-human monoclonal antibodies (mAbs) were used: Brilliant Violet (BV)421 anti-CD56 (NCAM 16.2), BV510 anti-CD3 (UCHT1), BV510 anti-CD14 (MΦP9), BV510 anti-CD19 (SJ25C1), BV510 anti-CD123 (9F5), PE anti-CD123 (9F5), PE anti-CD7 (M-T701) and PerCP-Cy5.5 anti-IFNγ (B27) from BD Biosciences; FITC anti-CD16 (B73.1) and APC anti-TNF (MAb11) from BioLegend; PE anti-CD300a (E59.126) and PE anti-2B4 (clone C1.7) from Beckman Coulter; PE anti-CD107a (REA792) and PE-Vio770 anti-NKp80 (4A4.D10) from Miltenyi Biotec; eFluor660 anti-Eomes (WD1928) from eBioscience. Dead cells were detected with the LIVE/DEAD™ Fixable Aqua Dead Cell Stain Kit for 405nm excitation from Invitrogen, following manufacturer's protocol.

The following reagents were also used: Foxp3/Transcription Factor Staining Buffer Set from eBioscience; Brilliant Stain Buffer, BD GolgiStop™ Protein Transport Inhibitor (monensin), BD GolgiPlug™ Protein Transport Inhibitor (brefeldin A) and BD Perm/Wash™ Buffer from BD Bioscience; and paraformaldehyde (PFA) from Sigma-Aldrich/Merck.

Methods Details

Peripheral blood mononuclear cell isolation.

Fresh PBMCs from healthy donors were obtained from buffy coats by Ficoll (GE Healthcare) density gradient centrifugation and cryopreserved in Fetal Bovine Serum (FBS) (GE Healthcare Hyclone) with 10% Dimethylsulfoxide (DMSO) (Thermo Scientific Scientific).

Flow cytometry: Phenotypical studies.

For phenotypical studies, cryopreserved PBMCs from healthy donors, HIV-1-infected subjects and multiple myeloma patients were thawed at 37ºC and washed twice with RPMI 1640 medium with L-Glutamine (Lonza). Then, cells were incubated for 1 hour at 37ºC with 10U DNase (Roche) in R10 medium (RPMI 1640 medium containing GlutaMAX from Thermo Fisher Scientific, 10% FBS and 1% Penicillin-Streptomycin from Thermo Fisher Scientific). Afterwards, cells were counted and washed with Phosphate Buffered Saline (PBS) (Gibco, Thermo Fisher Scientific). Then, dead cells were excluded by using the LIVE/DEAD reagent (Invitrogen, Thermo Fisher Scientific). For the staining of NK cell surface markers, cells were first washed with PBS containing 2.5% of Bovine Serum Albumin (BSA) (Millipore) and then incubated for 30 minutes at 4ºC with fluorochrome-conjugated mAbs. To identify NK cells, first viable cells that were negative for CD3, CD14 and CD19 were electronically gated, and then, by using the anti-CD56 mAb in combination with mAbs against CD16, NKp80, CD300a, 2B4 and/or CD7, NK cells were classified in three subsets: $CD56^{bright}$, $CD56^{dim}$ and $CD56^{neg}$. After this, cells were washed again with 2.5% BSA in PBS and fixed and permeabilized with Foxp3/Transcription Factor Staining Buffer Set (eBioscience, Thermo Fisher Scientific) following manufacturer's recommendations. Finally, cells were stained using anti-Eomes mAb for 30 minutes at room temperature (RT) and washed with Permeabilization Buffer 1x (eBioscience). Sample acquisition was carried out in a MACSQuant Analyzer 10 flow cytometer (Miltenyi Biotec).

Flow cytometry: Functional assays.

For functional assays, after DNase treatment, PBMCs from HIV-1-infected subjects and healthy donors were counted and plated at 0.5×10^6 cells/well in 48 well plates in NK cell culture medium (RPMI 1640 medium with GlutaMAX, 10% FBS, 1% penicillin streptomycin, 1% non-essential amino acids and 1% Sodium-Pyruvate). PBMCs were then primed with interleukin (IL)-15 (10ng/mL) and cultured for 20 hours. For cytokine stimulation, IL-12 (10ng/mL) and IL-18 (50ng/mL) were also added to plated PBMCs. For target cell stimulation, K562 cells were added after the 20 hours of culture in IL-15 at Effector:Target (E:T) 1:1 ratio (0.5×10^6 PBMCs and 0.5×10^6 K562 cells). Then, IL-12+IL-18 and K562 stimulated PBMCs were cultured for 6 hours. CD107a was added at the start of the co-culture period and protein transport inhibitors were added after 1 hour for the rest of the incubation time following manufacturer's protocol. Afterwards, viability and surface marker staining was performed as explained above. For intracellular staining, cells were fixed with 4% PFA for 15 minutes on ice and then washed twice with 2.5% BSA in PBS. After this, cells were permeabilized with BD Perm/Wash Buffer 1X for 15 minutes at RT. Finally, the corresponding mAbs were added for 30 minute and cells were washed with BD Perm/Wash Buffer 1X before acquisition in the MACSQuant Analyzer 10 flow cytometer (Miltenyi Biotec). The percentage of positive cells for CD107a, IFNγ and TNF was calculated after subtracting the non-stimulus condition.

Quantification and Statistical Analysis.

Data were analysed using FlowJo™ v10.4.1. GraphPad Prism v8.01 software was used for graphical representation and statistical analysis. As specified in all figure legends, each dot in the graphs represents a donor. Data were represented showing means ± standard error of the mean (SEM) or median as indicated in the figure legend. Prior to statistical analyses, data were tested for normal distribution with Kolmogórov-Smirnov normality test. In the case of multiple myeloma patients, an outlier was identified and removed using Grubb test (alpha=0.05). If data were normally distributed, t test for paired values was used to determine significant differences. Non-normal distributed data were compared with Wilcoxon matched-pairs signed rank test. Kruskal-Wallis test was used for multiple comparisons of non-normal data (Figure 4A). *p<0.05, **p<0.01, ***p<0.001, ****p<0.0001.

Appendix I: CoRIS Members

Executive committee
Santiago Moreno, Inma Jarrín, David Dalmau, Maria Luisa Navarro, Maria Isabel González, Federico Garcia, Eva Poveda, Jose Antonio Iribarren, Félix Gutiérrez, Rafael Rubio, Francesc Vidal, Juan Berenguer, Juan González, M Ángeles Muñoz-Fernández.

Fieldwork data management and analysis
Inmaculada Jarrin, Belén Alejos, Cristina Moreno, Carlos Iniesta, Luis Miguel Garcia Sousa, Nieves Sanz Perez, Marta Rava

BioBanK HIV Hospital General Universitario Gregorio Marañón
M Ángeles Muñoz-Fernández, Irene Consuegra Fernández

Hospital General Universitario de Alicante (Alicante)
Esperanza Merino, Gema García, Irene Portilla, Iván Agea, Joaquín Portilla, José Sánchez-Payá., Juan Carlos Rodríguez, Lina Gimeno, Livia Giner, Marcos Díez, Melissa Carreres, Sergio Reus, Vicente Boix, Diego Torrús

Hospital Universitario Central de Asturias (Oviedo)
Víctor Asensi, Eulalia Valle, María Eugenia Rivas Carmenado, Tomas Suarez-Zarracina Secades, Laura Pérez Is

Hospital Universitario 12 de Octubre (Madrid)
Rafael Rubio, Federico Pulido, Otilia Bisbal, Asunción Hernando, Lourdes Domínguez, David Rial Crestelo, Laura Bermejo, Mireia Santacreu
Hospital Universitario de Donostia (Donostia-San Sebastián)
José Antonio Iribarren, Julio Arrizabalaga, María José Aramburu, Xabier Camino, Francisco Rodríguez-Arrondo, Miguel Ángel von Wichmann, Lidia Pascual Tomé, Miguel Ángel Goenaga, Mª Jesús Bustinduy, Harkaitz Azkune, Maialen Ibarguren, Aitziber Lizardi, Xabier Kortajarena., Mª Pilar Carmona Oyaga, Maitane Umerez Igartua

Hospital General Universitario De Elche (Elche)
Félix Gutiérrez, Mar Masiá, Sergio Padilla, Catalina Robledano, Joan Gregori Colomé, Araceli Adsuar, Rafael Pascual, Marta Fernández, José Alberto García, Xavier Barber, Vanessa Agullo Re, Javier Garcia Abellan, Reyes Pascual Pérez, María Roca

Hospital General Universitario Gregorio Marañón (Madrid)
Juan Berenguer, Juan Carlos López Bernaldo de Quirós, Isabel Gutiérrez, Margarita Ramírez, Belén Padilla, Paloma Gijón, Teresa Aldamiz-Echevarría, Francisco Tejerina, Francisco José Parras, Pascual Balsalobre, Cristina Diez, Leire Pérez Latorre., Chiara Fanciulli

Hospital Universitari de Tarragona Joan XXIII (Tarragona)
Francesc Vidal, Joaquín Peraire, Consuelo Viladés, Sergio Veloso, Montserrat Vargas, Montserrat Olona, Anna Rull, Esther Rodríguez-Gallego, Verónica Alba., Alfonso Javier Castellanos, Miguel López-Dupla

Hospital Universitario y Politécnico de La Fe (Valencia)
Marta Montero Alonso, José López Aldeguer, Marino Blanes Juliá, María Tasias Pitarch, Iván Castro Hernández, Eva Calabuig Muñoz, Sandra Cuéllar Tovar, Miguel Salavert Lletí, Juan Fernández Navarro.

Hospital Universitario La Paz/IdiPAZ
Juan González-Garcia, Francisco Arnalich, José Ramón Arribas, Jose Ignacio Bernardino de la Serna, Juan Miguel Castro, Ana Delgado Hierro, Luis Escosa, Pedro Herranz, Víctor Hontañón, Silvia García-Bujalance, Milagros García López-Hortelano, Alicia González-Baeza, Maria Luz Martín-Carbonero, Mario Mayoral, Maria Jose Mellado, Rafael Esteban Micán, Rocio Montejano, María Luisa Montes, Victoria Moreno, Ignacio Pérez-Valero, Guadalupe Rúa Cebrián, Berta Rodés, Talia Sainz, Elena Sendagorta, Natalia Stella Alcáriz, Eulalia Valencia.

Hospital Universitari MutuaTerrassa (Terrasa)
David Dalmau, Angels Jaén, Montse Sanmartí, Mireia Cairó, Javier Martinez-Lacasa, Pablo Velli, Roser Font, Marina Martinez, Francesco Aiello

Hospital Universitario de La Princesa (Madrid)
Ignacio de los Santos, Jesus Sanz Sanz, Ana Salas Aparicio, Cristina Sarria Cepeda, Lucio Garcia-Fraile Fraile, Enrique Martín Gayo.

Hospital Universitario Ramón y Cajal (Madrid)
Santiago Moreno, José Luis Casado Osorio, Fernando Dronda Nuñez, Ana Moreno Zamora, Maria Jesús Pérez Elías, Carolina Gutiérrez, Nadia Madrid, Santos del Campo Terrón, Sergio Serrano Villar, Maria Jesús Vivancos Gallego, Javier Martínez Sanz, Usua Anxa Urroz, Tamara Velasco

Hospital General Universitario Reina Sofía (Murcia)
Enrique Bernal, Alfredo Cano Sanchez, Antonia Alcaraz García, Joaquín Bravo Urbieta, Angeles Muñoz Perez, Maria Jose Alcaraz, Maria del Carmen Villalba.

Hospital Nuevo San Cecilio (Granada)
Federico García, José Hernández Quero, Leopoldo Muñoz Medina , Marta Alvarez, Natalia Chueca, David Vinuesa García , Clara Martinez-Montes., Carlos Guerrero Beltran, Adolfo de Salazar Gonzalerz, Ana Fuentes Lopez

Centro Sanitario Sandoval (Madrid)
Montserrat Raposo Utrilla, Jorge Del Romero, Carmen Rodríguez, Teresa Puerta, Juan Carlos Carrió, Mar Vera, Juan Ballesteros, Oskar Ayerdi.

Hospital Universitario Son Espases (Palma de Mallorca)
Melchor Riera, María Peñaranda, Mª Angels Ribas, Antoni A Campins, Carmen Vidal, Francisco Fanjul, Javier Murillas, Francisco Homar., Helem H Vilchez, Maria Luisa Martin, Antoni Payeras.

Hospital Universitario Virgen de la Victoria (Málaga)
Jesús Santos, Crisitina Gómez Ayerbe, Isabel Viciana, Rosario Palacios, Carmen Pérez López, Carmen Maria Gonzalez-Domenec

Hospital Universitario Virgen del Rocío (Sevilla)
Pompeyo Viciana, Nuria Espinosa, Luis Fernando López-Cortés.

Hospital Universitario de Bellvitge (Hospitalet de Llobregat)
Daniel Podzamczer, Arkaitz Imaz, Juan Tiraboschi, Ana Silva, María Saumoy, Paula Prieto

Hospital Costa del Sol (Marbella)
Julián Olalla Sierra, Javier Pérez Stachowski., Alfonso del Arco, Javier de la Torre, José Luis Prada, José María García de Lomas Guerrero

Hospital General Universitario Santa Lucía (Cartagena)
Onofre Juan Martínez, Francisco Jesús Vera, Lorena Martínez, Josefina García, Begoña Alcaraz, Amaya Jimeno.

Complejo Hospitalario Universitario a Coruña (Chuac) (A Coruña)
Angeles Castro Iglesias, Berta Pernas Souto, Alvaro Mena de Cea.

Hospital Universitario Virgen de la Arrixaca (El Palmar)
Carlos Galera, Helena Albendin, Aurora Pérez, Asunción Iborra, Antonio Moreno, Maria Angustias Merlos, Asunción Vidal, Marisa Meca

Hospital Universitario Infanta Sofia (San Sebastian de los Reyes)
Inés Suárez-García, Eduardo Malmierca, Patricia González-Ruano, Dolores Martín Rodrigo, Mª Pilar Ruiz Seco.

Hospital Universitario Príncipe de Asturias (Alcalá de Henares)
José Sanz Moreno, Alberto Arranz Caso, Cristina Hernández Gutiérrez, María Novella Mena.

Hospital Clínico Universitario de Valencia (València)
María José Galindo Puerto, Ramón Fernando Vilalta, Ana Ferrer Ribera.

Hospital Reina Sofía (Córdoba)
Antonio Rivero Román, Antonio Rivero Juárez, Pedro López López, Isabel Machuca Sánchez, Mario Frias Casas, Angela Camacho Espejo

Hospital Universitario Severo Ochoa (Leganés)
Miguel Cervero Jiménez, Rafael Torres Perea

Nuestra Señora de Valme (Sevilla)
Juan A Pineda, Pilar Rincón Mayo, Juan Macias Sanchez, Nicolas Merchante Gutierrez, Luis Miguel Real, Anais Corma Gomez, Marta Fernandez Fuertes, Alejandro Gonzalez-Serna
